# Hierarchical Spinning of Janus Textiles with Anisotropic Wettability for Wound Healing

**DOI:** 10.34133/research.0129

**Published:** 2023-05-08

**Authors:** Han Zhang, Lingyu Sun, Jiahui Guo, Yuanjin Zhao

**Affiliations:** ^1^ Department of Rheumatology and Immunology, Nanjing Drum Tower Hospital, School of Biological Science and Medical Engineering, Southeast University, Nanjing, Jiangsu 210096, China.; ^2^ Oujiang Laboratory (Zhejiang Lab for Regenerative Medicine, Vision and Brain Health), Wenzhou Institute, University of Chinese Academy of Sciences, Wenzhou, Zhejiang 325001, China.

## Abstract

Wound healing and tissue repair are recognized as basic human health problems worldwide. Attempts to accelerate the reparative process are focused on developing functional wound dressings. Herein, we present novel Janus textiles with anisotropic wettability from hierarchical microfluidic spinning for wound healing. The hydrophilic hydrogel microfibers from microfluidics are woven into textiles for freeze-drying treatment, followed by the deposition of electrostatic spinning nanofibers composed of hydrophobic polylactic acid (PLA) and silver nanoparticles. The electrospun nanofiber layer can be well coupled with the hydrogel microfiber layer to generate Janus textiles with anisotropic wettability due to the roughness of the hydrogel textile surface and the incomplete evaporation of PLA solution when reaching the surface. For wound treatment with the hydrophobic PLA side contacting the wound surface, the wound exudate can be pumped from the hydrophobic to the hydrophilic side based on the wettability differential derived drainage force. During this process, the hydrophobic side of the Janus textile can prevent excess fluid from infiltrating the wound again, preventing excessive moisture and preserving the breathability of the wound. In addition, the silver nanoparticles contained in the hydrophobic nanofibers could impart the textiles with good antibacterial effect, which further promote the wound healing efficiency. These features indicate that the described Janus fiber textile has great application potential in the field of wound treatment.

## Introduction

As a natural barrier of human body, the skin executes important physiological functions to maintain homeostasis, such as protection, sensation, temperature regulation, absorption, secretion and excretion, metabolism, and immune regulation [1–4]. When suffering from trauma, abrasions, skin ulceration, or burns, the tissue structures would be damaged, or even be accompanied by serious complications after bacterial infection. To achieve rapid wound healing, many biomedical dressings including biofilms, porous sponges, and functionalized hydrogels have been developed in recent years [5–14]. Among them, hydrogels have attracted much attention because of their superior properties such as maintaining a moist wound healing environment, reducing the surface temperature of the wound, promoting cell proliferation and migration, as well as facilitating the diffusion and penetration of nutrients [15–22]. However, these hydrophilic hydrogel materials would stay overly wet after absorbing liquid, which is not conducive to the continuous absorption of exudate from wound surface. In addition, the swelling of hydrogels caused by liquid absorption makes it difficult for the wounds to realize sufficient oxygen exchange, thus hindering the process of wound healing. Therefore, a new type of hydrogel-based wound dressing is still highly anticipated.

**Fig. 1.**
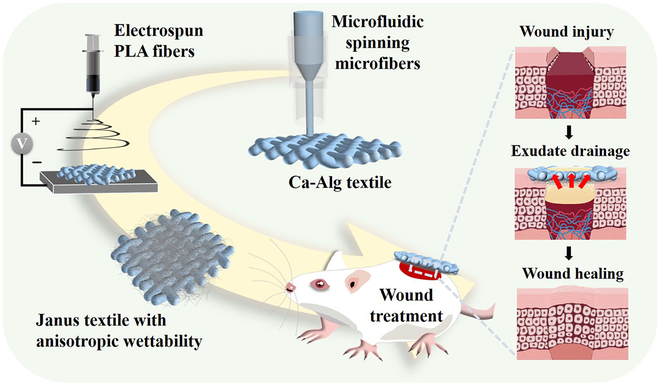
Scheme of the fabrication process and application. Schematic illustration of the hierarchical spinning fabrication process and the wound healing application of the Janus fibrous textile with anisotropic wettability.

In this paper, we proposed novel Janus textiles with anisotropic wettability for wound healing by using hierarchical microfluidic spinning technologies, as schemed in Fig. 1. Microfluidics is an emerging field that encompasses the intersection of chemistry, optics, biology, fluid mechanics, and micro-nano processing [23–29]. Taking advantage of the remarkable fluid manipulation capacity of microfluidics, a series of microfibers with different components, topography, and functions have been developed by microfluidic spinning [30–33]. When constructing hydrogel-based materials, these microfibers as building blocks could provide micron-scale morphological features for the system. In contrast, microfluidic electrospinning techniques are suitable for preparing fibers at the nanoscale [34–36]. The derived nanofibers possess mechanical, structural, and biological similarities to extracellular matrix, which could serve as medical dressings to offer the necessary support and guidance for cell proliferation and tissue growth. More importantly, the wettability of electrospinning nanofibers could be easily controlled, through choosing biomaterials with expected properties such as hydrophobic polymers. Therefore, we conceived that the integration of these 2 kinds of functional fibers at different scales would develop a novel Janus textile for promoting wound healing.

Herein, we prepared the desired wound dressing through combining microfluidic spinning and electrospinning techniques. Sodium alginate has been shown to have several advantages in the field of wound healing, including its ability to absorb water and form a gel-like substance, as well as its ability to promote cell proliferation and tissue regeneration. Based on the precise manipulation of a capillary microfluidic device, the calcium alginate (Ca-Alg) microfibers could be continuously generated and collected. During the process, the diameter of the microfibers could be accurately controlled by altering the flow rate ratio of the inner-phase Na-Alg and outer-phase CaCl_2_ solutions. Subsequently, the nanofibers composed of polylactic acid (PLA) doped with antibacterial silver nanoparticles (Ag NPs) were deposited on the freeze-dried hydrogel microfiber-woven textiles by utilizing an electrostatic spinning technique. Because of the roughness of the hydrogel textile surface and the incomplete evaporation of PLA solution when reaching the surface, the electrospun nanofiber layer could be well coupled with the hydrogel layer to form the Janus textiles with anisotropic wettability. When applied in animal experiments, the hydrophobic PLA side was faced down for wound treatment. Benefitting from the wettability difference, the wound exudate could be pumped from the hydrophobic side to the hydrophilic side under the effect of drainage force. In addition, the hydrophobic side of the Janus textile could block the excess fluid from infiltrating the wound again, thus maintaining the breathability of the wound surface. More importantly, the Ag NPs incorporated in the hydrophobic nanofibers exhibited an excellent antibacterial effect to further promote the wound healing process. These features make this Janus medical textile with anisotropic wettability ideal for tissue regeneration and other biomedical applications.

## Results

**Fig. 2.**
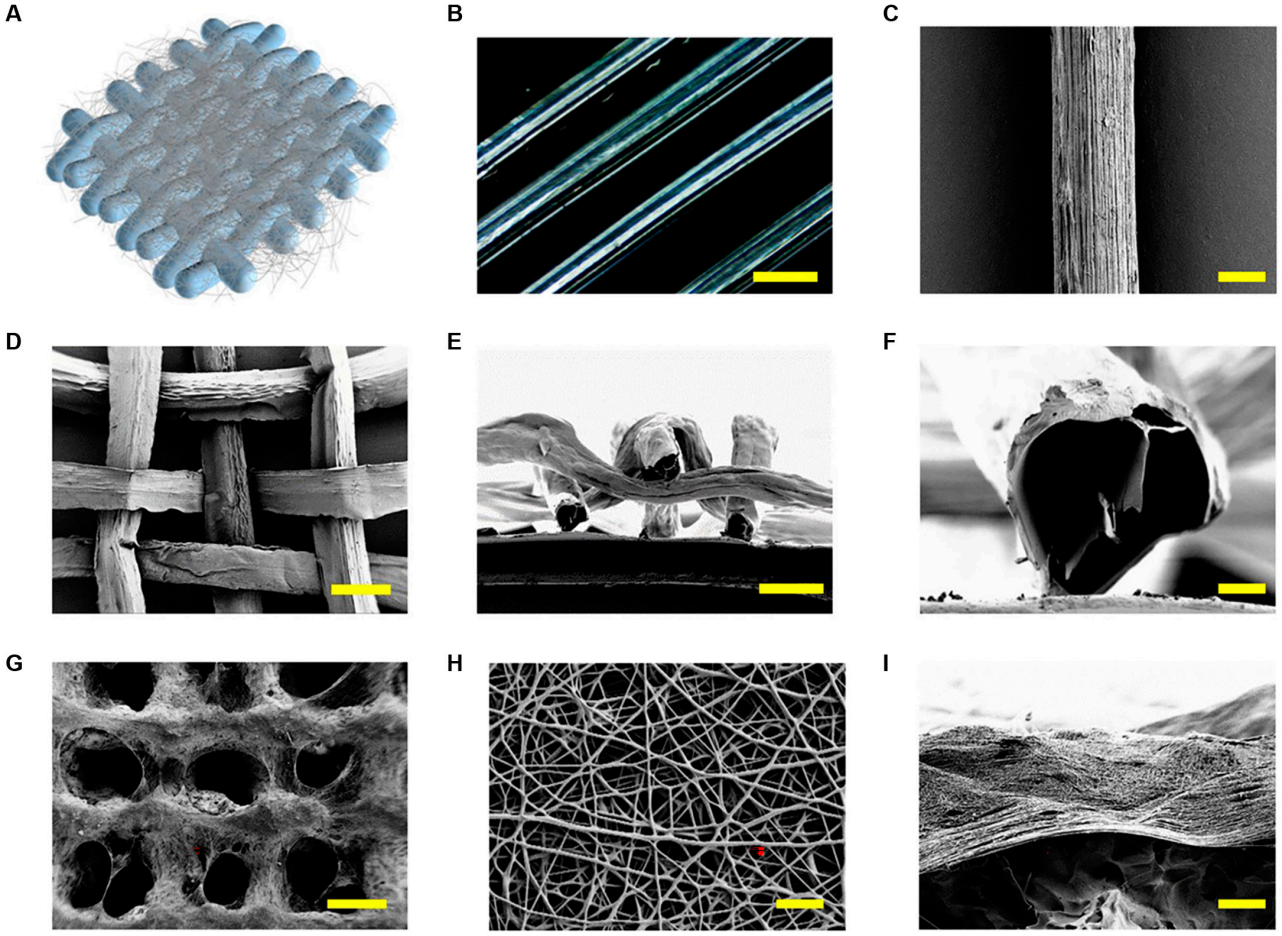
Characterization of the hydrogel microfibers and electrospinning PLA nanofibers. (A) The schematic diagram of the hierarchical-structured Janus fiber textile. (B) Microscope image of the Ca-Alg microfibers. The scale bar indicates 300 μm. (C) Scanning electron microscopy (SEM) images of the Ca-Alg microfiber. The scale bar is 100 μm. (D and E) SEM images of the Ca-Alg microfiber woven textile at (D) surface and (E) cross-section. The scale bars are 200 μm. (F) The magnified field of the cross-section. The scale bar is 100 μm. (G and H) SEM image of the Janus fiber textile (G) and the magnified field of the electrospinning PLA nanofibers (H). The scale bars are 200 and 5 μm, respectively. (I) The junction of the Ca-Alg microfibers and PLA nanofibers. The scale bar is 50 μm.

In a typical experiment, a single-emulsion microfluidic device was used to create Ca-Alg microfibers. Calcium chloride solution was injected into the outer phase, and sodium alginate solution was injected into the inner phase of a capillary tube with a conical output. Due to instantaneous ion exchange, homogeneous Ca-Alg microfibers could be continuously generated online, as shown in Fig. S1A. By altering the inner- and outer-phase flow rates, the diameter of Ca-Alg microfibers could be accurately manipulated. It was discovered that when the inner phase's flow rate increased and the outer phase's flow rate decreased, the fiber's diameter gradually increased (Fig. S1B). The Ca-Alg microfibers produced at the same flow rate were uniform in size and shape, as shown in Fig. 2B and C. Subsequently, we simply wove the prepared microfibers, and after freeze-drying, it could be found that this microfiber hydrogel fabric had a porous structure throughout the surface and inside (Fig. 2C to E), which enabled the Ca-Alg microfiber textile dressing to achieve a better water absorption effect and could be in line with the current development trend of medical wound dressings.

In general, these hydrophilic hydrogel dressings are generally beneficial for loading various growth factors. However, excessive hydrophilicity owns many disadvantages, such as leaving the wound surface in an overly moist state, resulting in the retention of exuded fluid from the wound surface, which greatly increases the risk of wound infection and hinders subsequent wound healing process. Therefore, we designed a novel Janus dressing based on hydrophilic Ca-Alg microfiber textile. As shown in Fig. 2A, G, and H, a layer of uniform PLA nanofibers was combined with the freeze-dried Ca-Alg microfiber textile by electrostatic spinning. Since the volatilization of organic solvent took some time, the PLA nanofibers could be closely combined with the Ca-Alg microfiber textile, as shown in Fig. 2I. By adjusting the peristaltic pump's flow rate and the high-voltage power supply's voltage, the size of the PLA nanofibers could be readily controlled. As the flow rate increased and the voltage decreased, the diameter of nanofibers gradually increased (Fig. S2). Medical wound dressings with good water vapor transmission rate can provide a good breathing environment for new cells during wound healing, so as to achieve the effect of promoting wound healing. In addition, materials with good swelling properties indicate that they have a strong ability to absorb water molecules and other substances from the microenvironment, which is conducive to the transfer and exchange of nutrients required for cell growth and the excretion of cell metabolites. The study of the air permeability and swelling properties of the Ca-Alg microfiber textile and the hierarchical-structured Janus fiber textile in Fig. S3 showed that the addition of PLA nanofibers did not affect the properties of the dressing itself, and Janus fiber textiles still achieved good air permeability and absorption, meeting the basic requirements of wound dressings.

Subsequently, a specific study of the wettability of the Janus fiber textile was carried out. It could be seen from Fig. S4 that the Ca-Alg microfiber side had a good hydrophilic effect, while the PLA nanofiber side had a good hydrophobic effect. In addition, it has also revealed that no liquid remained on the surface of PLA nanofiber side after the removal of the fluorescent droplets, confirming the excellent antisoiling effect of the PLA textile, which could ensure the cleanliness of the textile on the side in contact with the wound. To verify the stability of the wettability of the PLA fabric side, we measured the contact angle of 3 different liquids on the fabric surface for 7 d using deionized water, fluorescein isothiocyanate-bovine serum albumin solution, and rhodamine B solution to simulate different body fluids. The results demonstrated that the water contact angle values of PLA side decreased slightly after 7 d, but it still maintained good hydrophobicity, confirming that PLA nanofibers would maintain good stain resistance after prolonged contact with the wound.

**Fig. 3.**
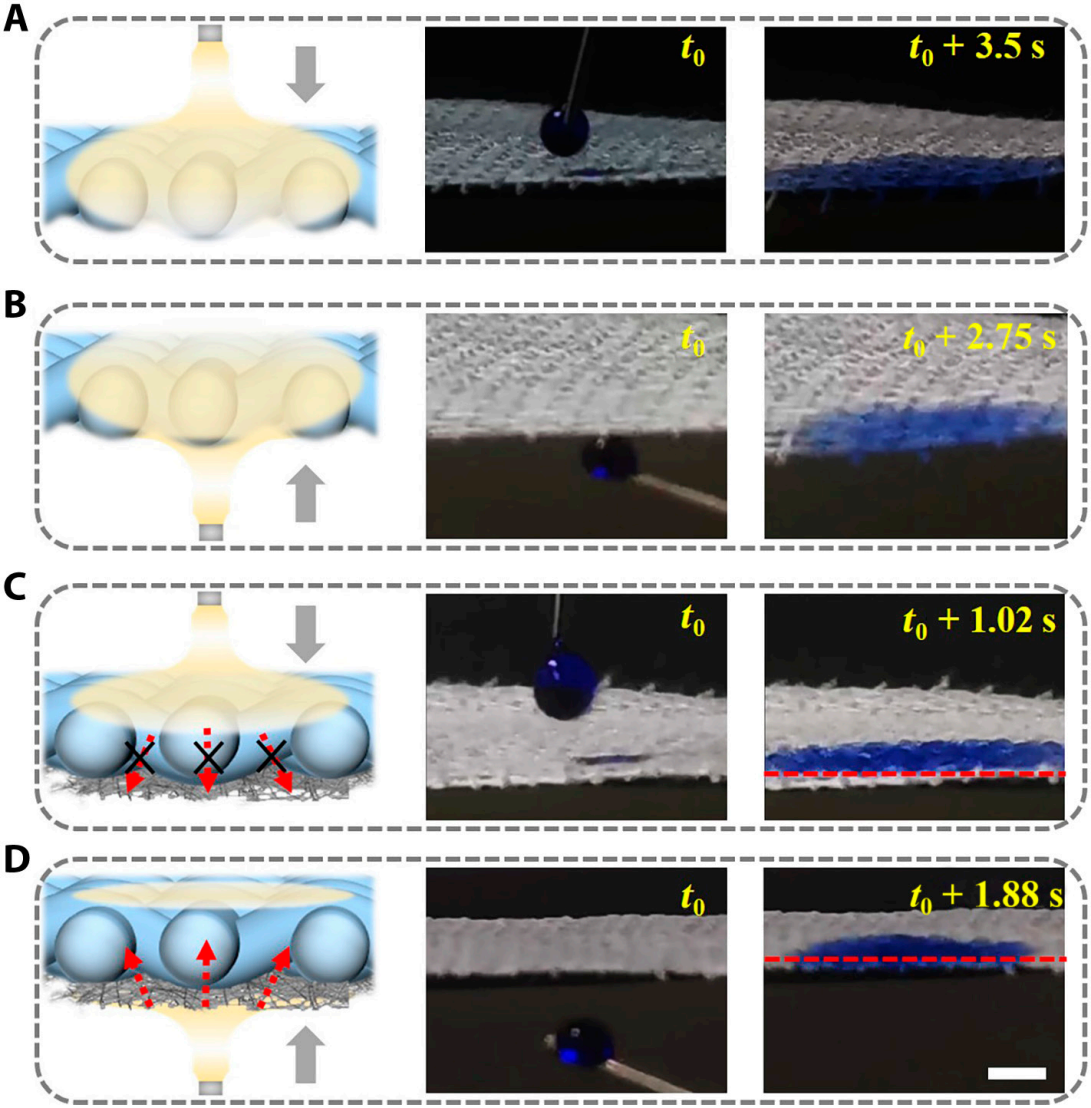
Demonstration of unidirectional fluid draining capability of the Janus fiber dressing by comparing with the Ca-Alg fiber textile dressing. (A to D) Schematic and images of the droplet interacting with Ca-Alg fiber textile dressing (A and B) and the Janus fiber dressing (C and D). The scale bar is 3 mm.

To further investigate the enhancement of the Janus structure on the performance of the dressing, we conducted a liquid absorption study on the Ca-Alg microfiber textile and the hierarchical-structured Janus fiber textile using dyed droplets. As can be seen from Fig. 3, both sides of the Ca-Alg microfiber textile had good absorption of liquid and the whole dressing was wetted. In the case of Janus textile dressing, when the droplet touched the hydrophilic portion, the droplet rapidly wetted the hydrophilic portion but was blocked by the hydrophobic layer. In addition, the dye droplets were injected into the hydrophobic layer and moistened the hydrophilic portion upon contact with the hydrophobic layer. As the solution continued to drip out, the liquid discharge process proceeded unidirectionally from the hydrophobic side to the hydrophilic side. This confirmed that the hierarchical-structured Janus fiber textile not only could achieve the water absorption effect of ordinary dressings but also ensure the relative dryness of the trauma surface through unidirectional liquid delivery, solving the problem of excessive wetting.

**Fig. 4.**
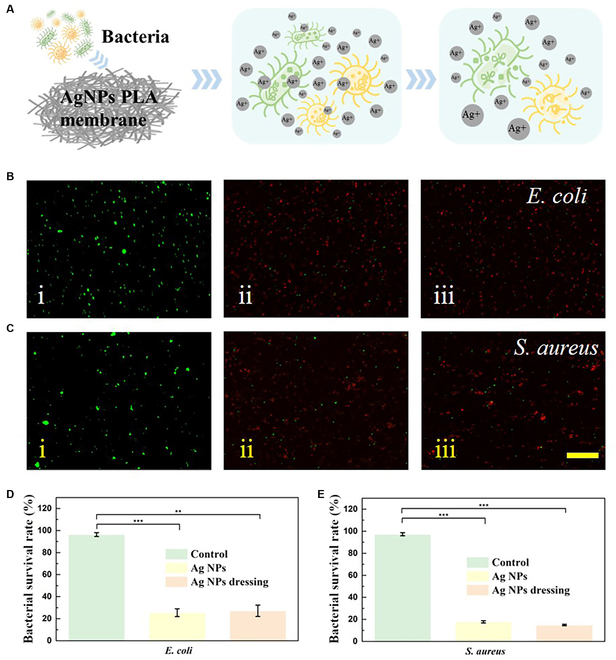
Antibacterial property of the Janus fibrous membrane. (A) Schematic illustration of anti-infective activity. (B and C) Fluorescent staining images of *S. aureus* and *E. coli* cocultured with PBS solution, Ag NPs, and the Janus fiber textile, respectively. The scale bar is 50 μm. (D and E) Death rate of *S. aureus* and *E. coli* in 3 groups. NS, not significant; **P < 0.01, ***P < 0.001.

In order to endow the hierarchical-structured Janus fiber textile with more functions, we additionally added Ag NPs to the PLA solution. Ag NPs are antibacterial due to their ability to interact with bacterial cells and disrupt their structure and metabolism. Ag NPs are highly reactive and able to interact with bacterial cells at the nanoscale due to their high surface-area-to-volume ratio and high surface energy. This mechanism is different from that of antibiotics, which often target specific cellular processes, and thus may be less prone to the development of resistance. The release of silver ions (Ag^+^) upon contacting with bacterial cells is one of the primary mechanisms through which Ag NPs exert their antibacterial activity. Ag^+^ ions are capable of penetrating bacterial cell walls and binding to thiol groups on proteins and enzymes, thereby disrupting their function and causing cell death (Fig. 4A). It could be seen from the energy-dispersive spectrum analysis that Ag NPs were evenly dispersed in PLA solution (Fig. S5). Subsequently, we tested the antibacterial property of PLA nanofiber dressing loaded with Ag NPs. *Escherichia coli* and *Staphylococcus aureus* were chosen as 2 representative bacteria for the studies. The bacterial staining results showed that both bacteria survived and maintained their intrinsic morphology in the phosphate-buffered saline (PBS) control. In contrast, most of the bacteria died when cocultured with Janus fiber textiles, indicating that Janus fiber textiles can break the cell membrane, leading to efflux and accumulation of nucleic acids (Fig. 4B and C). The statistical results also showed that more than 70% of *S. aureus* and *E. coli* cocultured with Janus fiber textiles died (Fig. 4D and E). In addition, Ag NPs alone and Ag NPs-loaded PLA textiles exhibited similar antibacterial properties compared to the blank group. This demonstrated that the antibacterial performance of Ag NPs before and after encapsulation was not affected and the same antibacterial effect could be achieved in the distributed PLA textiles. We can conclude that the excellent antimicrobial activity of Ag NPs confers competitive potential to Janus fiber textile patches in avoiding complications associated with bacterial infections and promoting wound healing.

**Fig. 5.**
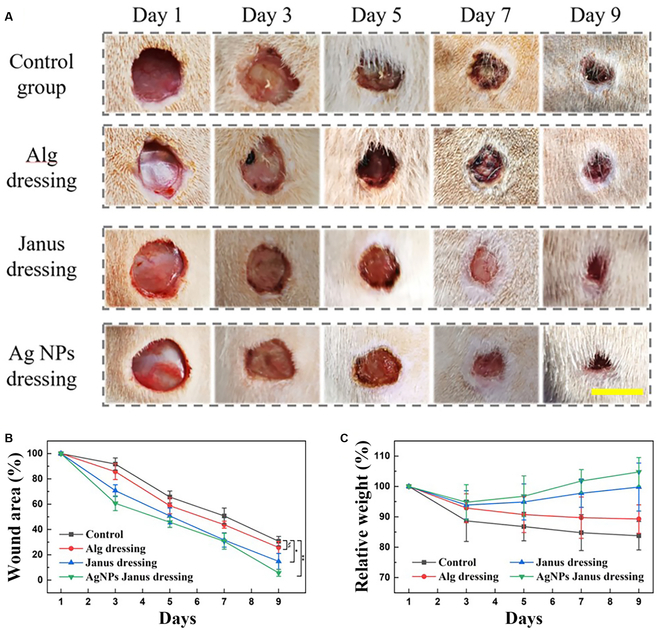
The healing process of different groups. (A) Images of wounds on day 0, day 3, day 5, day 7, and day 9 from different groups: the PBS control group, the Alg dressing group, the Janus dressing group, and Ag NPs dressing group. The scale bar is 1 cm. (B) The wound healing rate in different groups. (C) The relative body weight of different groups. *P < 0.05, **P < 0.01, ***P < 0.001.

To determine the practical value of Janus textile dressing loaded with Ag NPs in the healing of infected wounds, we conducted in vivo tests subsequently. Prior to the practical application of Janus dressings to animals, the biocompatibility of the textile dressings was investigated. 3T3 cells were cocultured with 3 different textiles. The daily cell status was recorded, and Cell Counting Kit-8 (CCK-8) experiments were used to assess specific data (Fig. S6). In the remaining 3 groups, there was no decrease in cellular activity compared to the control group; consequently, these textiles could be studied in vivo. On the back of mice, a 1-cm-diameter model of a *Staphylococcus aureus*-infected full-thickness skin defect was built. The test samples were separated into 4 groups consistent with biocompatibility experiments: Control, Alg dressing-treated, Janus dressing-treated, and Ag NPs Janus dressing-treated. These groups' wound healing capacity and wound closure processes were meticulously documented and evaluated. Figure 5 showed that the wounds of the mice treated with Janus textile dressing loaded with Ag NPs were largely healed after 9 d. In contrast, healing of wounds treated with the plain textile dressing required more time and was inferior to that of the Janus dressing and marginally superior to the control group. The wounds treated by Janus textile dressing loaded with Ag NPs required noticeably less healing time and were in better physical condition than the other groups, according to quantitative analysis of wound closure rate and body weight over time. The Janus textile dressing's capacity to carry excessive exudate unidirectionally may account for its capacity to facilitate wound healing in vivo. In addition, the Ag NPs added to the textile also demonstrated good antibacterial effect, which further promoted wound healing efficiency.

**Fig. 6.**
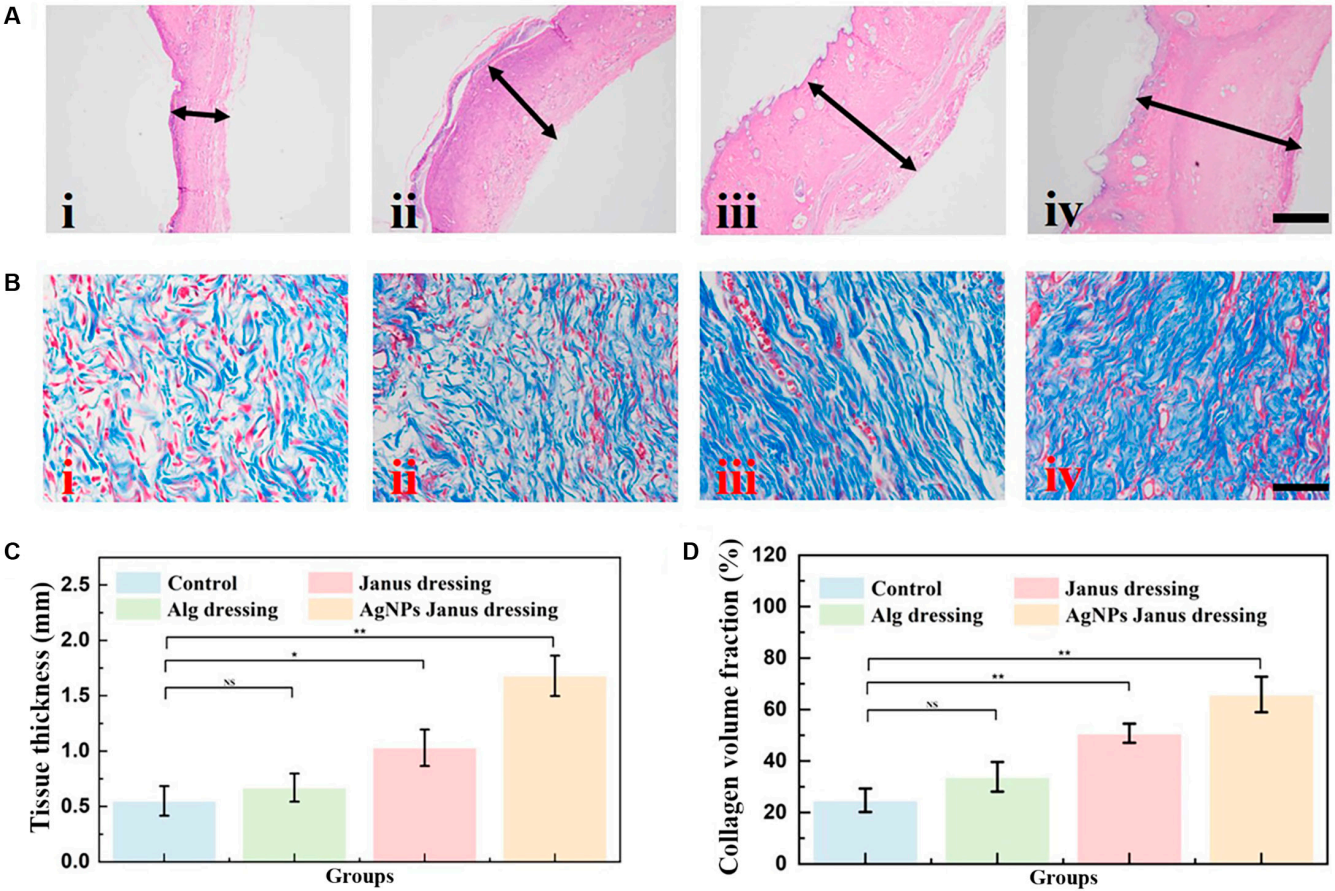
The results of HE staining and Masson staining. (A) Representative HE staining of wounds after 9 d. The scale bar is 500 μm. (B) Representative Masson staining of wounds after 9 d. The scale bar is 50 μm. (C and D) The statistical analysis of HE (C) and Masson (D) staining. *P < 0.05, **P < 0.01.

The recovery of each group was then investigated using imaging of the regenerated granulation tissue stained with hematoxylin and eosin (HE), as well as the relative thickness of granulation tissue in each group. In comparison to the control group and the Ca-Alg microfiber textile group, the degree of tissue regeneration in the Janus textile dressing group and the Janus textile loaded with Ag NPs group was considerably higher. In the control group, the granulation tissue was 0.55 mm. The tissue thickness of the Alg dressing-treated group, Janus textile dressing-treated group, and Ag NPs Janus textile dressing-treated group were 0.67, 1.03, and 1.69 mm, respectively, as shown in Fig. 6. Compared with the other groups, the control group had poorer wound healing because no additional protective measures were taken. The Alg microfiber textile group was also much less effective in healing than the other 2 groups because it was not loaded with active factors to promote wound healing. The Janus textile dressing treatment group dressing could transfer excess exudate unidirectionally, hence reducing the risk of infection and keeping the wound dry. Therefore, the wound healing was relatively good in the Janus fiber textile group, and due to the presence of Ag NPs, the Ag NPs Janus textile dressing treatment group presented the best healing results. Furthermore, we also observed that, the Ag NPs Janus textile dressing treatment group had the highest collagen deposition, which was highly aligned with the histology analysis findings (Fig. 6B). These studies also reveal the superior wound healing capability of functionalized Janus textile dressings.

Interleukin-6 (IL-6), tumor necrosis factor-α (TNF-α), and CD31 are all key players in the complex process of wound healing. IL-6 and TNF-α are both proinflammatory cytokines that can promote the recruitment of immune cells to the site of injury and stimulate the proliferation and migration of cells involved in tissue repair. The results of IL-6 and TNF-α also showed that the Ag NPs Janus textile dressing treatment group presented the least inflammatory factors, which also confirmed the good healing of the wounds. To further investigate the healing effect, we assessed the extent of angiogenesis. Vascularization plays a crucial role in wound repair by supplying oxygen and nutrients to the wound site and removing metabolic waste products. The formation of new blood vessels, also known as angiogenesis, is a critical step in wound healing. During the inflammatory phase of wound healing, blood vessels in the injured area dilate to allow immune cells to reach the site of injury. This increase in blood flow helps to remove debris and bacteria from the wound. In wound healing process, CD31 is involved in angiogenesis, the formation of new blood vessels that supply oxygen and nutrients to the healing tissue. Microscopic observations based on CD31 staining (Fig. S7) revealed that the Ag NPs Janus textile dressing treatment group had increased intratrauma bed vascular density. Thus, accelerated angiogenesis can provide sufficient nutrition and oxygen and accelerate the migration of humoral factors and essential cells to the trauma, which in turn promotes collagen and granulation tissue production, thus facilitating wound healing. The results showed that the Janus textile dressing is an ideal wound-healing dressing that transports excess exudate unidirectionally.

## Discussion

To conclude, we proposed a novel hierarchical spinning strategy for the preparation of Janus fiber textiles with anisotropic wettability. The advantages of sodium alginate and PLA in promoting cell proliferation and tissue regeneration in wound repair have been fully utilized. Compared with ordinary gauze, this fiber textile could also achieve better water absorption effect and could be loaded with various active factors and drugs to promote the repair process. Besides, with the combination of hydrophilic and hydrophobic fibers on 2 different sides and the junction of hydrophilic and hydrophobic sites, this textile could achieve unidirectional fluid transport, thus draining the exuded fluid from the wound surface and avoiding excessive wetting. In addition, to give more functions to this Janus fiber textile, we added Ag NPs on the hydrophobic PLA side, and upon contact with the wound surface, the Ag NPs can effectively achieve the antibacterial effect, further improving the efficiency of wound repair. We believe that these Janus fiber textiles with anisotropic wettability are ideal for biomedical applications.

## Materials and Methods

### Materials

Sodium alginate (Alg) and calcium chloride (CaCl_2_) were purchased from Sinopharm Chemical Reagent Co., Ltd. Fluorescein isothiocyanate-bovine serum albumin was obtained from Beijing Zhongke Chenyu Technology Co., Ltd. Propidium iodide, SYTO 9 Green, CCK-8, and calcein were provided by Shanghai Beyotime Biotechnology Co., Ltd. PLA was brought from Jinan Daigang Biomaterial Co., Ltd. Ethyl acetate was acquired from Shanghai Aladdin Biochemical Technology Co., Ltd. PBS, Dulbecco’s modified Eagle medium, fetal bovine serum, trypsin, and penicillin–streptomycin were purchased from Gibco. All the chemical reagents were used as received.

### Preparation of Janus textiles

The Ca-Alg microfibers were prepared by a microfluidic chip whose inner-phase solution was sodium alginate solution and outer-phase solution was calcium chloride solution. Subsequently, these microfibers were weaved into 2 cm × 2cm medical textiles. For better hydrophilicity and liquid absorption, these textiles were freeze-dried overnight. The resulting porous textiles served as the base material for the Janus textiles. The porous textiles were placed on the aluminum foil paper that was connected to the negative electrode of the high-voltage power supply, and the syringe containing the PLA solution was connected to the positive electrode of the high-voltage power supply. The receiving distance was fixed at 10 cm. When the voltage reached a certain value, the electrospinning process started. The time of electrospinning was different in different experiments. Generally speaking, the time was controlled at more than 3 min to ensure that the surface of the Ca-Alg textiles could be covered with PLA nanofibers. By adjusting the flow rate of the PLA solution and the voltage value of the high-voltage power supply, we could obtain PLA fibers with different diameters.

### Permeability test

The lysis bottle was filled with 5 ml of deionized water, and a cut dressing was affixed to the bottle's mouth. It should be emphasized that the bottle's seal must be maintained. The sealed lysis bottle was placed on the balance, and its mass was measured as *M*_0_ (g). It was then placed in a constant temperature shaker at 37 °C for days (t) before being removed and its mass was measured as *M* (g) on the balance. The water vapor transmission rate of the dressing can be estimated by measuring the mass change twice and using the following formula: *W* = (*M*_0_ − *M*)/(st). The “s” represented the opening area of the lysis bottle.

### Swelling test

The dressings were trimmed down to a 20 mm × 20 mm sample to be measured, and the trimmed sample was placed in a balance and its mass was measured as *M*_0_. The trimmed sample was then placed in a centrifuge tube with 25 ml of PBS solution, after which the tube was placed in a constant temperature shaker at 37 °C. After weighing, the sample was put back into the tube, and PBS solution was added to the tube to make the volume still 25 ml. After that, the centrifugal tubes were put into the shaker for testing. The swelling properties were calculated as follows: *M* (%) = (*M*_t_ − *M*_0_)/*M*_0_ × 100.

### Biocompatibility of the Janus textiles

The experiments were divided into 4 groups: blank control group, plain microfiber textile group, Janus textile group, and Ag NPs-loaded Janus textile group, each with a cell density of 2 × 10^5^ cells/ml. The plain microfiber textile group, Janus textile group, and Ag NPs-loaded Janus textile group were fully immersed in the medium to reach lysis equilibrium to avoid differences in the volume of the medium during the experiment, and the textiles were irradiated with ultraviolet light overnight to achieve sterilization. The cover glasses were used to press down on the materials to ensure that the hydrophobic materials were completely immersed in the medium. Cocultures were incubated for 0, 1, 2, and 3 d for CCK-8 assay.

### Antibacterial test in vitro

Gram-positive *Staphylococcus aureus* and Gram-negative *Escherichia coli* were selected as representative strains to verify the antibacterial activity of AgNPs-loaded Janus dressings. The 2 bacteria were cultured according to McFarland standard until the turbidity of bacterial suspension was about 0.5. Subsequently, the bacteria were collected and resuspended in PBS buffer. The experiment was divided into 3 groups: blank control group, AgNPs group, and AgNPs Janus fiber dressing group. The 3 groups were incubated in 24-well plates for 24 h and stained with live-dead dyes (SYTO 9 and propidium iodide), and the fluorescence images were observed by fluorescence microscopy and analyzed numerically.

### Preparation of wound infection model

A model for acute wound infection was developed. All Sprague Dawley rats were sedated by intraperitoneal injection after being fasted for the previous night. On each rat's back, a circular full-thickness cutaneous wound measuring 1 cm × 1 cm was made, and 200 ml of *S. aureus* solution containing 1 × 10^8^ CFU/ml was then injected into the wounds. The rats were then randomized into 4 groups at random with 6 rats in each group: Control (washed by PBS), Alg microfiber textile group, Janus textile-treated group, and AgNPs Janus fiber textile dressing group. The animals were returned to their own cages for unrestricted access to food and water, and the rate of healing of their wounds was monitored every 2 d. The granulation tissue above the incision was removed from the sacrificed rats after 9 d. After the wound has healed, the dressings were removed manually. For immunohistochemical and histological investigation, the granulation tissue was submerged in neutral formaldehyde at a 10% concentration.

## Data Availability

The data are available from the authors upon a reasonable request.

## Supplementary Materials

Supplementary Materials Fig. S1. Continuous formation of Ca-Alg microfibers and the relationship between the microfiber diameter and flow rate. Fig. S2. The relationship between the diameter of the PLA nanofibers and voltage flow rate. Fig. S3. The permeability and swelling rate of Alg fiber textile and Janus fiber textile. Fig. S4. The water contact angle on Alg fiber dressing and electrospinning PLA nanofibers. Fig. S5. The energy-dispersive spectrum (EDS) analysis of the electrospinning membrane side. Fig. S6. The biocompatibility of the dressings. Fig. S7. Immunostaining for IL-6, TNF-α, and CD31 staining for collagen in different groups.
