# Supplementary material for: Hierarchical Spinning of Janus Textiles with Anisotropic Wettability for Wound Healing
Source: Research (Wash D C). 2023 May 8;6:0129. doi: 10.34133/research.0129 (PMC10202375; doi:10.34133/research.0129)
Supplement: Supplementary Materials — Fig. S1. Continuous formation of Ca-Alg microfibers and the relationship between the microfiber diameter and flow rate. Fig. S2. The relationship between the diameter of the PLA nanofibers and voltage flow rate. Fig. S3. The permeability and swelling rate of Alg fiber textile and Janus fiber textile. Fig. S4. The water contact angle on Alg fiber dressing and electrospinning PLA nanofibers. Fig. S5. The energy-dispersive spectrum (EDS) analysis of the electrospinning membrane side. Fig. S6. The biocompatibility of the dressings. Fig. S7. Immunostaining for IL-6, TNF-α, and CD31 staining for collagen in different groups. [file research.0129.f1.docx]

Supplementary Materials


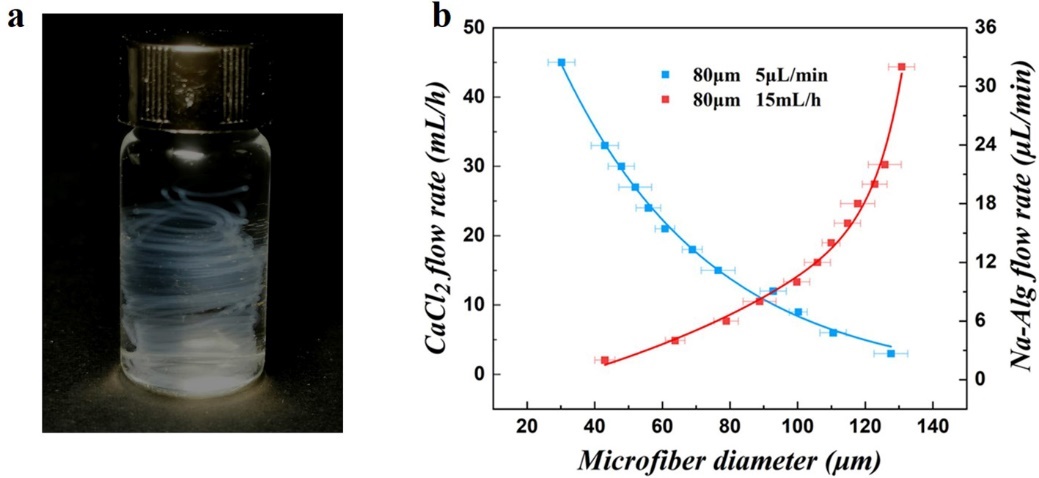


**Figure S1.** **The continuous generation of microfibers and the influence parameters of the diameter.** a) The optical image of continuous formation of Ca-Alg microfibers. b) The relationship between the microfiber diameter and flow rate.


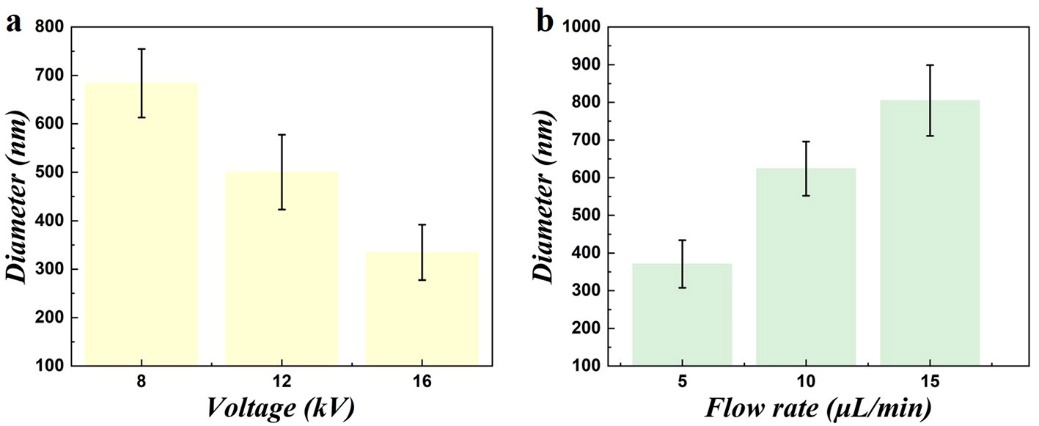


**Figure S2.** **The relationship between the diameter of the electrospinning PLA nanofibers and voltage a), flow rate b)**.


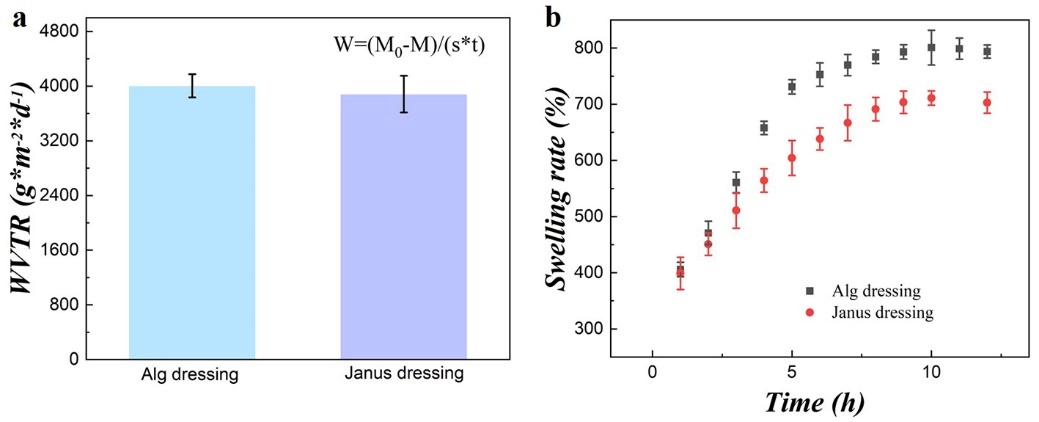


**Figure S3.** **The permeability a) and swelling rate b) of Alg fiber textile and Janus fiber textile.**


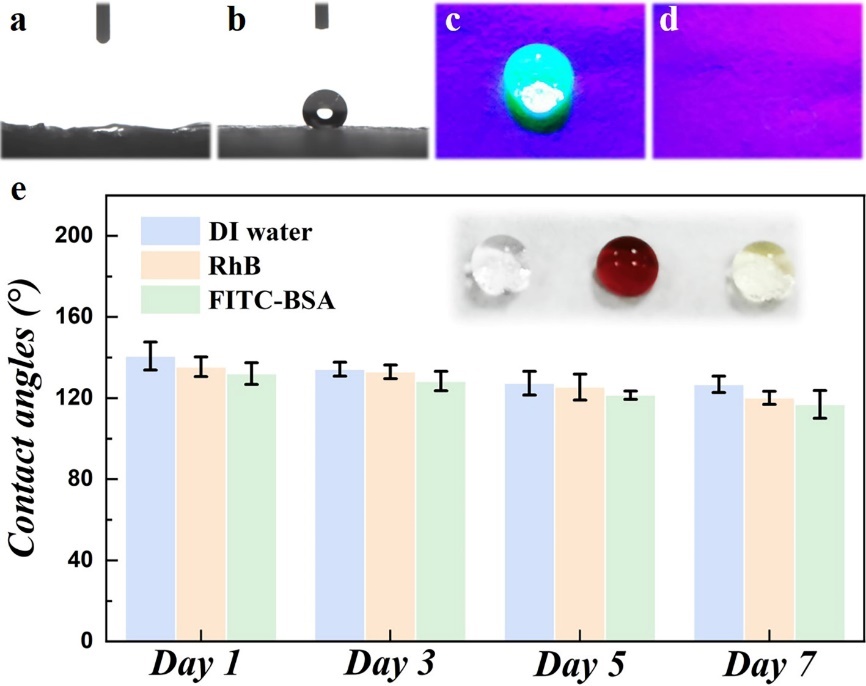


**Figure S4. The wettability of the Janus textile.** a, b) The water contact angle on Alg fiber dressing a) and electrospinning PLA nanofibers b). c, d) Images before c) and after d) removal of fluorescent droplets on the electrospun fiber membrane. e) Contact angle stability of three simulated liquids.


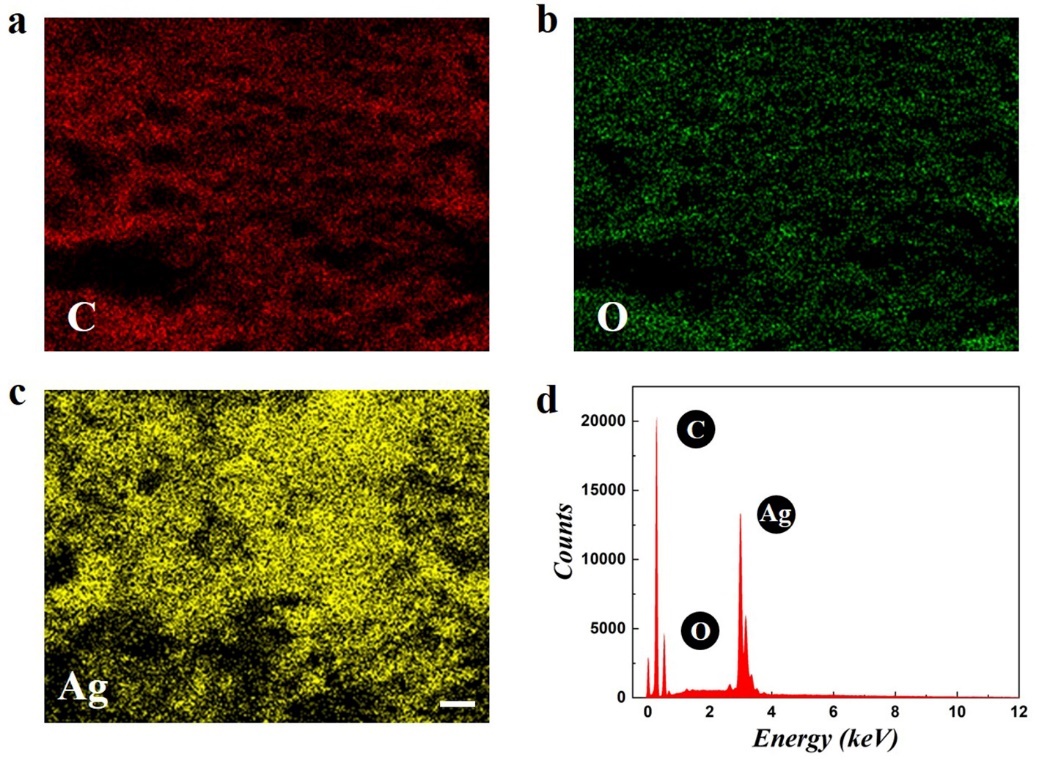


**Figure S5.** **The energy dispersive spectrum (EDS) analysis of the electrospinning membrane side of the Ag NPs loaded Janus fiber dressing.** The scale bar is 10 μm.


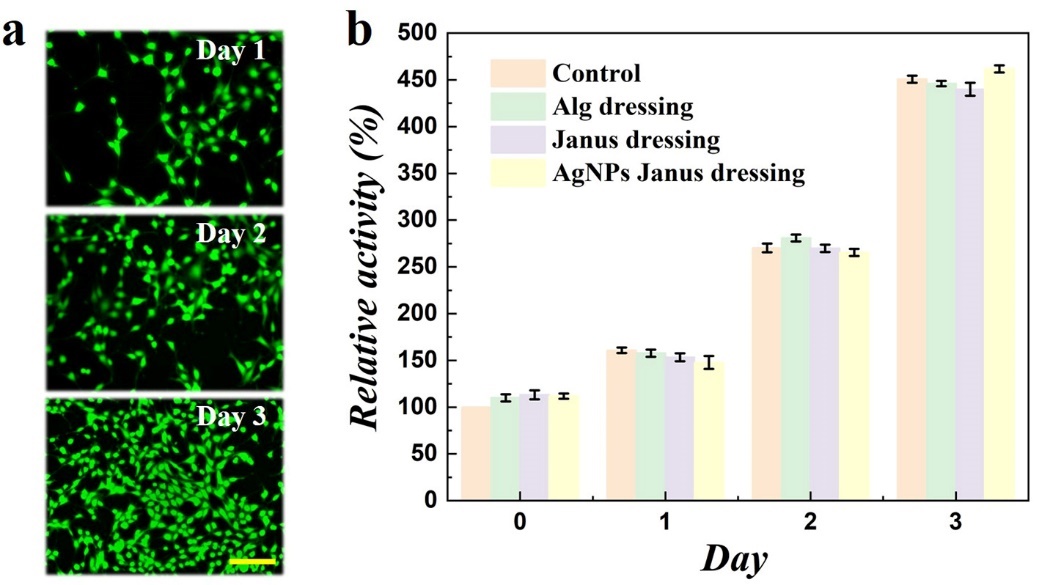


**Figure S6. The biocompatibility of the dressings.** a) The fluorescent images of 3T3 cells on Day 1, Day 2, and Day 3 in AgNPs Janus dressing group. b) The relative activity of 3T3 cells analyzed via CCK8. The scale bar is 200 μm.


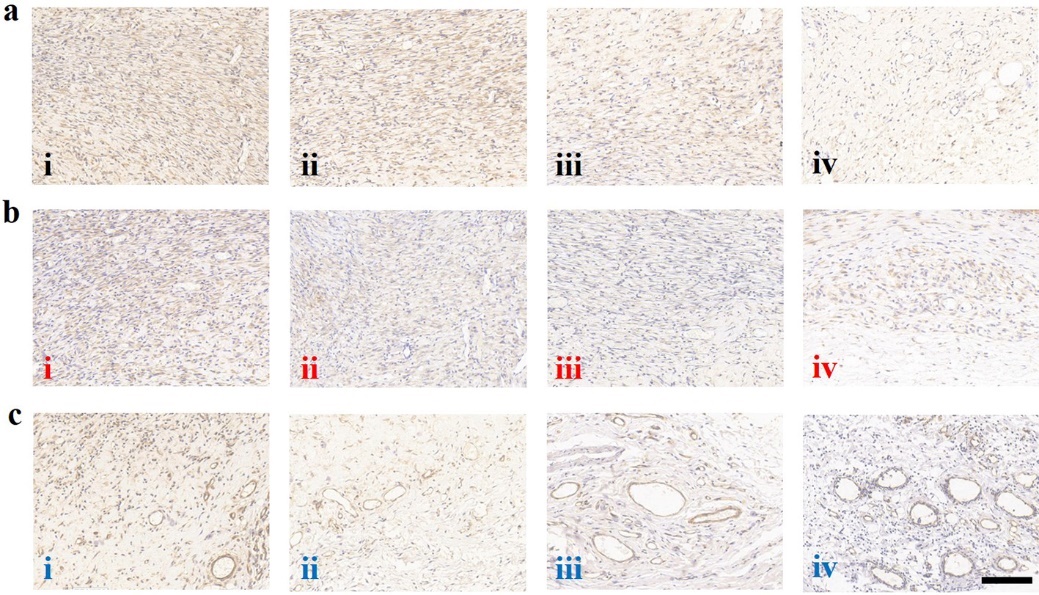


**Figure S7.** **Immunostaining for a) IL-6, b) TNF-α and c) CD31 staining for collagen in different groups.** The scale bar is 100 μm.
